# Novel histone deacetylase inhibitors and embryo aggregation enhance cloned embryo development and ES cell derivation in pigs

**DOI:** 10.1371/journal.pone.0204588

**Published:** 2018-09-27

**Authors:** Chawalit Siriboon, Tzai-Shiuan Li, Chao-Wu Yu, Ji-Wang Chern, Jyh-Cherng Ju

**Affiliations:** 1 Graduate Institute of Biomedical Sciences, China Medical University, Taichung, Taiwan; 2 Department of Animal Science, Faculty of Agriculture, Ubon Ratchathani University, Ubon Ratchathani, Thailand; 3 Translational Medicine Research Center, China Medical University Hospital, Taichung, Taiwan; 4 School of Pharmacy, National Taiwan University, Taipei, Taiwan; 5 Department of Bioinformatics and Medical Engineering, Asia University, Taichung, Taiwan; 6 Department of Animal Science, National Chung Hsing University, Taichung, Taiwan; Peking University Third Hospital, CHINA

## Abstract

The histone deacetylase inhibitor (HDACi) has been investigated for treating cancers and many other diseases as well as enhancing the reprogramming efficiency in cloned embryos for decades. In the present study, we investigated the effects of two novel HDAC inhibitors, *i*.*e*., HDACi-14 and -79, at the concentrations of 0, 1, 2, or 4 μM on the development of embryos cloned by the oocyte bisection cloning technique (OBCT). Blastocyst rates for the reconstructed embryos reached 60% in the 2 μM HDACi-14-treated groups, which was higher (*P <* 0.05) compared to the untreated group (36.9%). Similarly, HDACi-79 treatment at 2 and 4 μM also conferred higher (*P <* 0.05) blastocyst rates than that of the untreated group (79.4, 74.2, and 50.0%, respectively). Both HDACi-14 and -79 treatments had no beneficial effect on total cell numbers and apoptotic indices of cloned embryos (*P >* 0.05). Histone acetylation profile by both HDACi-14 (2 μM) and -79 (2 μM) treatments demonstrated a drastic increase (*P <* 0.05) mainly in two-cell stage embryos when compared to the control group. After seeding on the feeder cells, the aggregated cloned blastocysts produced by the HDACi-79 treatment showed a significant increase of primary outgrowths compared to the control group (60.0% *vs*. 42.9%; *P <* 0.05). Finally, the cloned embryo-derived ES cell lines from aggregated cloned embryos produced from the HDACi-79-treated, HDACi-14-treated and control groups were established (5, 3, and 2 lines, respectively). In conclusion, the novel histone deacetylation inhibitors improve blastocyst formation and potentially increase the derivation efficiency of ES cell lines from the cloned porcine embryos produced *in vitro*. Depending on the purposes, some fine-tuning may be required to maximize its beneficial effects of these newly synthesized chemicals.

## Introduction

Embryonic stem (ES) cells represent a population of pluripotent cell type capable of self-renewal and differentiation into all somatic cell lineages. Meanwhile, somatic cell nuclear transfer (SCNT) has been envisioned as one promising way of generating personalized ES cells from specific somatic cells, which can be a valuable tool for clinical applications to study disease mechanisms, cell therapies, and even xenotransplantation [1–3]. In previous studies, ES or ES-like cells have also been established from SCNT derived embryos in various species such as mice, rabbits, bovine, primates, and pigs [3–9]. The establishment of ES cell lines in domestic animals could potentially impact studies of mammalian embryogenesis and regenerative medicine. Also, ES cells can be used to produce transgenic animals, such as pigs, serving as xenotransplantation donors. Pigs are also a desirable species to create pluripotent cell lines because of their immunological, physiological and functional similarities to humans [10]. The morphology of porcine ES cell colony is also similar to that of human ES cells which is more like flattened cell aggregates rather than dome-shaped colonies (as in murine species).

The establishment of porcine ES cell lines from the inner cell mass (ICM) of SCNT blastocysts has not been well demonstrated. Limited success in derivation of ES cell lines from SCNT embryos (ntES cells) are mainly attributed to the poor efficiencies of SCNT, due to the compromised quality and low cell numbers of developing embryos that, in turn, contain fewer ES cell progenitors [11]. Histone acetylation in preimplantation embryos is essential for normal embryonic development [12–14]. Increased histone acetylation (hyperacetylation) is associated with the accessibility of DNA replication protein complexes or synthasomes to the entire genome that facilitates cell proliferation [15].

For cell reprogramming, it is generally accepted that many HDAC inhibitors function to inhibit HDACs by interfering with the removal of acetyl groups from histone proteins (reversible or irreversible). Many known HDACi, such as TSA, scriptaid, SAHA, oxamflatin, valproic acid (VPA) have been used to assist reprogramming of cloned embryos, with the most popular one being TSA, which inhibits Classes I and II HDAC families [16]. Several studies have shown an improved development of cloned embryos when being incubated with TSA during early embryonic stages [17–22]. Previous studies in bovine SCNT have also shown that treatment of donor cells or reconstructed embryos with TSA enhances blastocyst rates [18,19]. Prather and colleagues reported that Scriptaid treatment significantly enhanced the development of porcine SCNT embryos to the blastocyst stage which dramatically increased the cloning efficiency [23]. In addition, the blastocyst rates and the total cell number of cloned buffalo blastocysts are improved by Scriptaid treatment [24]. SAHA and oxamflatin could also improve the full-term development of cloned mouse embryos without discernible abnormalities [25]. However, valproic acid has been reported as having little or no effect on mouse cloning efficiency [25,26]. In contrast, Huangfu et al. demonstrated that valproic acid increased the reprogramming efficiency of mouse fibroblasts by more than 100-fold for the establishment of induced pluripotent stem (iPS) cells [27].

Embryo aggregation to improve embryo development *in vitro* has been reported in some studies [8,28–32]. Lee et al. demonstrated that blastocyst rates, total cell numbers, and Oct4 expression were all improved by aggregation of porcine embryos [29]. Similarly, we previously demonstrated that embryo aggregation improved developmental competency and the ratio of ICM cells to total cell numbers in cloned porcine embryos [30]. Although some claimed that aggregations of cloned mice embryos did not improve embryo development, the increased total cell numbers and Oct4 expression levels in the aggregated cloned embryos were clearly demonstrated [28,32].

In this study, we aimed to improve the efficacies of embryo cloning and ES cell derivation by treating aggregated cloned embryos with our novel histone deacetylation inhibitors, i.e., HDACi-14 and HDACi-79. This is the first attempt of using these newly synthesized drugs on early embryogenesis in cloned embryos.

## Materials and methods

### Animal use and ovary collection

Ovaries were collected from a local abattoir where pigs were slaughtered via electric shock under the guidelines enforced by governmental standard operation procedure. This study was carried out in strict accordance with the recommendations in the Institutional Animal Care and Use Committee (IACUC). The protocol was approved by the Committee on the Ethics of Animal Experiments of the National Chung Hsing University (permit number: 97–95).

### Preparation of somatic donor cells for nuclear transfer

Fibroblasts derived from ear biopsy of postnatal miniature piglets were cultured in Dulbecco’s modified Eagle medium (DMEM) supplemented with 10% FBS at 39°C in a 5% CO_2_ incubator. Somatic donor cells of passages 3–5 were cryopreserved in the DMEM medium supplemented with 30% FBS and 10% dimethylsulfoxide (DMSO) in liquid nitrogen until use.

After thawing, cells were grown for one week in DMEM supplemented with 10% FBS. Using 80–90% confluency, the cells were starved in DMEM supplemented with a low level of FBS (0.25%) for three days. Before nuclear transfer, cells were washed once with Dulbecco’s phosphate-buffered saline (DPBS) and subjected to trypsinization for 5 min, and then they were re-suspended in HEPES-buffered TCM-199 containing 20% FBS (T20). The fibroblast suspension was allowed to stand at 4°C until fusion with recipient cytoplasts.

### Oocyte preparation

Cumulus-oocyte complexes (COCs) were aspirated from follicles (2–6 mm in diameter) of slaughterhouse ovaries. Harvested COCs were subjected to *in vitro* maturation (IVM) for 42 h at 39°C with 5% CO_2_. The maturation medium was TCM 199 supplemented with 10% porcine follicular fluid, 10% FBS and gonadotropins (10 IU/mL hCG and 10 IU/mL PMSG).

After 42 h of IVM, cumulus cells were removed off matured (MII) oocytes by repeatedly pipetting in a 500 μL DPBS droplet containing hyaluronidase (1 mg/mL). Matured oocytes with a first polar body (PB) were selected and kept in a 100 μL droplet of HEPES-buffered TCM-199 supplemented with 10% FBS (T10) at 39°C under 5% CO_2_ until use.

### Embryo cloning procedures and culture

In this study oocyte bisection cloning technique (OBCT) was used as described previously [30]. Matured oocytes with 1^st^ PB were incubated in pronase solution (3.3 mg/ml) and then in HEPES-buffered TCM 199 supplemented with 33% FBS for 15–20 sec, subjected to partial digestion of the zona pellucida by rinsing in T10. After incubation in pronase solution, oocytes with partially digested but still visible zona pellucida were lined up in a 40 μL T10 droplet (20 oocytes in each droplet) containing cytochalasin B (2.5 mg/mL). Oocytes were rotated with a fire-polished glass pipette to identify the 1^st^ PB for oriented bisection with a microblade (ESE020, Bioniche Animal Health, USA, INC.) under a stereomicroscope. Less than half (20–30% in volume) of the ooplasm close to the PB protrusion was removed from the remaining ooplast. The demi-ooplasts without zona pellucida and chromatin or DNA were rinsed twice in T10 medium before fusion.

Cell fusion was performed by using a two-step protocol with two consecutive electrical fusions. First, the enucleated ooplast was transferred to the HEPES-TCM-199 droplet containing 1 mg/mL phytohaemagglutinin (PHA) for 5 sec, and then transferred into the T10 droplet that holds the donor cells (fibroblasts). Each ooplast was then released and carefully dropped on one fibroblast cell to allow pairing. The ooplast-fibroblast pairs were incubated in the fusion medium (0.3 M mannitol and 0.01% PVA) for 20 sec, and then transferred to the fusion chamber (with two electrodes separated by 1 mm). With an AC pulse (0.6 kV/cm), the cell pairs were aligned to the wire with the fibroblasts farthest from the wire. Fusion was then performed with one DC pulse (2.0 kV/cm) for 9 μsec. The ooplast-fibroblast pairs were then transferred from the fusion chamber to the T10 medium drop and incubated for 1–2 h before the second fusion.

The second fusion for the remaining ooplasts with the fused ooplast-fibroblast pairs was performed by first transferring the above ooplasts and pairs to the activation medium droplet (0.3 M mannitol, 0.1 mM MgSO_4_, 0.1 mM CaCl_2_ and 0.01% PVA) for equilibration. For fusion and initial activation, they were aligned (0.6 kV/cm AC) with the fused pairs farthest from the wire followed by a DC pulse (0.85 kV/cm, 80 μsec); then the ooplast-fibroblast triplets were incubated in T10 medium for 20 min to allow complete fusion prior to chemical activation with 6-DMAP for 4 h at 39°C in a 5% CO_2_ incubator. Thereafter, the porcine zygote medium-3 (PZM-3) supplemented with 3 mg/mL BSA was used for embryo culture. After parthenogenetic activation, embryos were washed four times with 200 μL PZM-3 and then cultured individually for 7 days in the WOW system covered with mineral oil at 39°C in a 5% CO_2_ incubator [30].

### Treatment with the HDAC inhibitor (HDACi)

HDACi-14 and HDACi-79, previously designated as, JWC022214 and JWC017379, respectively, were synthesized by Prof. Ji-Wang Chern’s laboratory, School of Pharmacy, National Taiwan University, Taipei, Taiwan [33]. HDACi-14, (E)-3-(2-ethyl-7-flouro-4-oxo-3-phenethyl-3,4-dihydroquinazolin-6-yl)-N-hydroxyacrylamide, with a molecular weight of 381.4 kD (C_12_H_20_FN_3_O_3_). HDACi-79, (E)-N-hydroxy-3-(4-oxo-3-phenethyl-3,4-dihydroquinazolin-7-yl), is C_19_H_17_N_3_O_3_ and a molecular weight of 335.36. Both drugs were synthesized to be structurally similar to hydroxamic acids to be more efficient in inhibiting of Class II HDACs, being effective even at lower nanomolar concentrations *in vitro*.

Preparation and dilution of HDACi (JWC022214 and JWC017379) stock solutions were performed by dilution of HDACi powder in DMSO. The HDACi stocks were kept at -20°C until used. Twenty min after the second fusion, cloned embryos were randomly distributed and transferred into PZM-3 medium containing 2 mM 6-DMAP and various concentrations of HDACi (0, 1, 2, and 4 μM) for 4 h at 39°C in 5% CO_2_. After chemical activation, embryos were repeatedly washed for four times through small droplets of PZM-3, and then transferred to the WOW system incubated with HDACi for an additional 18–20 h. The culture medium was replaced with PZM-3 without HDACi after incubation.

### Detection of DNA damage by TUNEL staining

The TUNEL labeling was based on manufacture protocols from our previous studies [22,30]. Briefly, cloned blastocysts at day 7 after nuclear transfer were harvested from each treatment group and rinsed three times with DPBS containing 0.1% polyvinylpyrrolidone. Embryos were fixed in fixative (4% paraformaldehyde/DPBS, v/v) for 24 h and then made permeable by 0.1% Triton X-100 in 0.1% citrate solution for 1 h at room temperature. Thereafter, embryos were incubated in the TUNEL reaction medium (*in situ* cell death detection kit, TMR red; Roche, Mannheim, Germany) for 1 h at 38.5°C in the dark. In the process, broken DNA ends of embryonic cells were labeled with TdT and fluorescein-dUTP. After incubation, embryos were washed in DPBS/BSA (DPBS + 0.1% bovine serum albumin) and finally mounted with DAKO fluorescence mounting medium (S3023, Dako North America, Inc., California, USA) containing Hoechst 33342 on glass slides and then were observed under an epifluorescence microscope (Nikon, Tokyo, Japan). The numbers of total cells and apoptotic cells were determined. The apoptotic index was calculated as follows:

Apoptoticindex=(No.ofTUNEL‑positivenuclei/totalNo.ofnuclei)×100

### Detection of histone acetylation in cloned embryos

Detection of histone acetylation was performed as described previously [22]. Briefly, OBCT cloned embryos were collected at 2-cell, 4-cell, and blastocyst stages from each treatment group (30 cloned embryos per group). After being fixed in 4% paraformadehyde (in DPBS) at 4°C for 1 h, embryos were then washed with DPBS containing 0.1% Tween-20 for 30 min and subsequently made permeable with 0.1% TritonX-100 in DPBS overnight at 4°C. They were washed again with 0.1% Tween-20 for 30 min and then incubated in 2% BSA (in DPBS) for 1 h at RT to block non-specific binding. The first primary antibody, rabbit against acH3 at Lys9/Lys14 (Cat #9677, Cell Signaling Technology Inc., Beverly, MA), was prepared by 1:300 dilution in 2% BSA-DPBS. Embryos were incubated with the primary antibody for 1 h and then washed with 0.1% Tween-20 for 30 min. Secondary antibody (mouse against histone H3, Santa Cruz Biotechnology, INC) was prepared at 1:200 dilution in 2% BSA-DPBS. Subsequently, embryos were incubated with the secondary antibody for 1 h and washed again with 0.1% Tween-20 for 30 min. After washing, embryos were incubated with the first secondary antibody against rabbit Ig G (Alexa Flour 488-conjugated, Invitrogen, Carlsbad, CA, USA; 1:200) for 1 h at RT. Embryos were further washed with 0.1% Tween-20 in DPBS for 30 min followed by incubating with the second secondary against mouse Ig G (Alexa Flour 555-conjugated, Invitrogen; 1:200) for 1 h at RT. After being washed with 0.1% Tween-20 in DPBS for 30 min, embryos were mounted on slides with 50% glycerol in DPBS. For the negative control, all primary antibodies were omitted from the staining protocol. To visualize the immunofluorescent labeling of acH3K9/K14 and total H3 in each treatment, embryos were observed under an epifluorescence microscope (Nikon, Tokyo, Japan). The average fluorescence intensity was measured by ImageJ software (Image Processing and Analysis in Java, Version 1.42, National Institutes of Health, Bethesda, MD, USA) for five random regions of the nucleoplasm (excluding nucleolar regions) after background subtraction and averaged the ratio of acH3K9/K14 to the total H3 signals per embryo.

### RNA purification, reverse transcription and quantitative real-time PCR

The quantification of all gene transcripts was performed as described previously [22,30]. In brief, 30 Day-7 cloned embryos of various treatments were collected and stored in lysis buffer at -80°C until used. Commercial TRIzol Reagent (Invitrogen) and RNeasy Mini Kit (Qiagen) were used for the extraction and purification of total RNA, respectively. Thereafter, purified RNA was quantified using a ND-1000 spectrophotometer (Nanodrop Technology, Wilmington, DE, USA) and qualitatively analyzed by Bioanalyzer 2100 (Agilent Technology, USA). The cDNA pool was prepared from each RNA sample; total RNA (19 ng) was reversely transcribed by using MMLV reverse transcriptase (Pro-mega), and the resulted products were diluted (1:2000) with DNase-free water. Each cDNA pool was stored at -20°C pending for real-time PCR analysis.

Real-time PCR was performed on the Roche LightCycler Instrument 1.5 using LightCycler FastStart DNA MasterPLUS SYBR Green I kit (Roche Cat. 03 515 885 001). The 10 mL reaction mixture contained a 2 μL master mix, 2 μLof 0.75 μM and 2 μL of 0.50 μM forward primer and reverse primer in 5% DMSO, respectively, and 6 μL cDNA sample solution. Sample analysess were performed in triplicates. The RT-PCR was programmed sequentially as 95°C for 10 min, 50 cycles of 95°C for 10 sec, 60°C for 15 sec, and 72°C for 10 sec. At the end of each RT-PCR run, a melting curve analysis was performed and data were automatically analyzed by the system. An amplification plot was generated for each cDNA sample. From each plot, the LightCycler3 Data Analysis Software (Roche Diagnostics) automatically calculated the CP value (crossing point, the turning point corresponds to the first maximum of the second derivative curve), which implies the beginning of an exponential amplification. The comparative CT method was applied for quantification of gene expression levels, as described previously [34]. The fold change of gene expression was calculated using the formula 2-(ΔΔCT Exp-Control), where

ΔΔCTExp=CTtarget‑CTrefofExpsample

ΔΔCTControl=CTtarget‑CTrefofcontrolsample

Primer sequences, GenBank accession numbers, and expected product sizes for the real-time PCR analysis are presented in Table 1.

**Table 1:** Primer sequences, GenBank accession numbers and expected amplicon sizes for qRT-PCR analyses.

Target genes	Primers	Sequences (5' to 3')	GenBank accession number	Product size (bp)
Oct4	Forward	CGCAACGAGAGGATTTTGAG	TC_168415	68
	Reverse	CGCCAGAGGAAAGGATACTG		
Nanog	Forward	TGCCTGGTGAACGCTTCTGGAA	NM_001129971	104
	Reverse	TGGTTGCTCCAAGACTGGCTGT		
Sox2	Forward	AGAAGAACAGCCCAGACCGAGTT	NM_001123197	108
	Reverse	CCGAGTTGTGCATCTTGGGGTT		
Cdx2	Forward	CCTCTCGCCCACAAATGTTCAC	TC_206866	82
	Reverse	TCCAACCGCACCTGTCTTTACC		
Bcl-xL	Forward	GCAGGTATTGAACGAACTCTTCCG	AJ_001203	107
	Reverse	GCATCTCCTTGTCTACGCTCTCC		
Bax	Forward	CTACCAAGAAGTTGAGCGAGTGTC	AJ_606301	85
	Reverse	ACGGCTGCGATCATCCTCTG		
β-actin	Forward	CCACGCCATCCTGCGTCT	AK_237086	168
	Reverse	CCATCTCCTGCTCGAAGTCCAG		

### Preparation of MEF feeders

Mouse fetal fibroblasts (MEFs) were isolated from day 13.5 fetuses *post- coitus*. The head, legs, and internal organs were removed by forceps and surgical scissors in a 3-mL droplet containing 1% ABAM/PBS. After washing twice with 1% ABAM/PBS, the carcass was minced into small pieces with a surgical blade. Minced fetal tissues were washed by centrifugation (300×g for 10 min) and subsequently seeded into culture dishes containing DMEM supplemented with 10% FBS at 37°C for 5–7 days. Derived MEF cells were cultured in DMEM medium supplemented with 10% FBS and sub-cultured when cells proliferated up to 80–90% confluency. Once 80% confluency was reached at passages 3 and 4, MEFs were treated with 10 μg/mL mitomycin-C (Sigma-Aldrich, MO, USA) for 3 h to arrest mitosis. After incubation, cells were washed several times with DPBS, and re-plated (8 × 10^4^ cells/well) in gelatin-coated, 4-well culture dishes.

MEFs feeders were inactivated 24 h before plating blastocysts or passaging pluripotent cell lines. Two h before use, culture medium was removed from feeder cells and replaced with porcine ES (pES) cell medium.

### Culture conditions for embryonic outgrowths

Quality cloned embryos for derivation of porcine ntES cells were produced by multiple embryo aggregations as described previously [8]. After parthenogenetic activation, reconstructed embryos were washed three times with 200 μL PZM-3. Cloned embryos were cultured individually for 2 days (with 20 h of HDACi treatment) up to the 4-cell stage in the WOW system covered with mineral oil at 39°C in an incubator containing 5% CO_2_ in air. Aggregation with three embryos (3×) was performed with the 4-celled embryos and the aggregated embryos were cultured continuously for an additional 5 days.

On day 7 cloned blastocysts were transferred from the culture dish and seeded onto a 4-well dish containing feeder layers in the pES cell medium, based on DMEM/F12 medium supplemented with 1 mM L-glutamine (Sigma-Aldrich), 0.1 mM β-mercaptoethanol (Sigma-Aldrich), 10 mM MEM non-essential amino acids (Sigma-Aldrich), antibiotics (50 units/mL penicillin G, and 50 mg/mL streptomycin sulfate: Invitrogen), 5 ng/mL basic FGF, 20 ng/mL human recombinant LIF, 10 μM ROCK inhibitor (Y-27632; 1596–5; Biovision, Milpitas, CA, USA), and 20% knockout serum replacement (KSR, Gibco, CA, USA). Blastocysts (day 7) were plated and cultured upto 8–10 days at 38.5 ºC in a 5% CO_2_ incubator and the medium was replaced with fresh pES cell medium on the second day after plating. When the diameter of colonies expanded to its full dimension, primary outgrowths were mechanically removed using a sterile glass pipette, and then were transferred to a 100 μL drop of fresh medium for cutting into small pieces (30–50 cells per piece) with a microblade (ESE020, Bioniche Animal Health, Inc., Pullman, WA, USA) and were mechanically pipetted under a stereomicroscope. Pieces of cell clusters were then passaged and cultured on freshly prepared MEF feeders.

### Statistical analysis

All the percentile data were arcsine transformed before performing analysis of variance (ANOVA). The ANOVA were tested by using the General Linear Model (GLM) procedure in SAS version 9 (SAS Institute, Cary NC, USA), followed by Tukey’s test to identify the treatment effects, and the difference between treatment groups at *P <* 0.05 was considered significant.

## Results

### Effects of HDACi-14 and HDACi-79 on the development of cloned pig embryos

**Fig 1:**
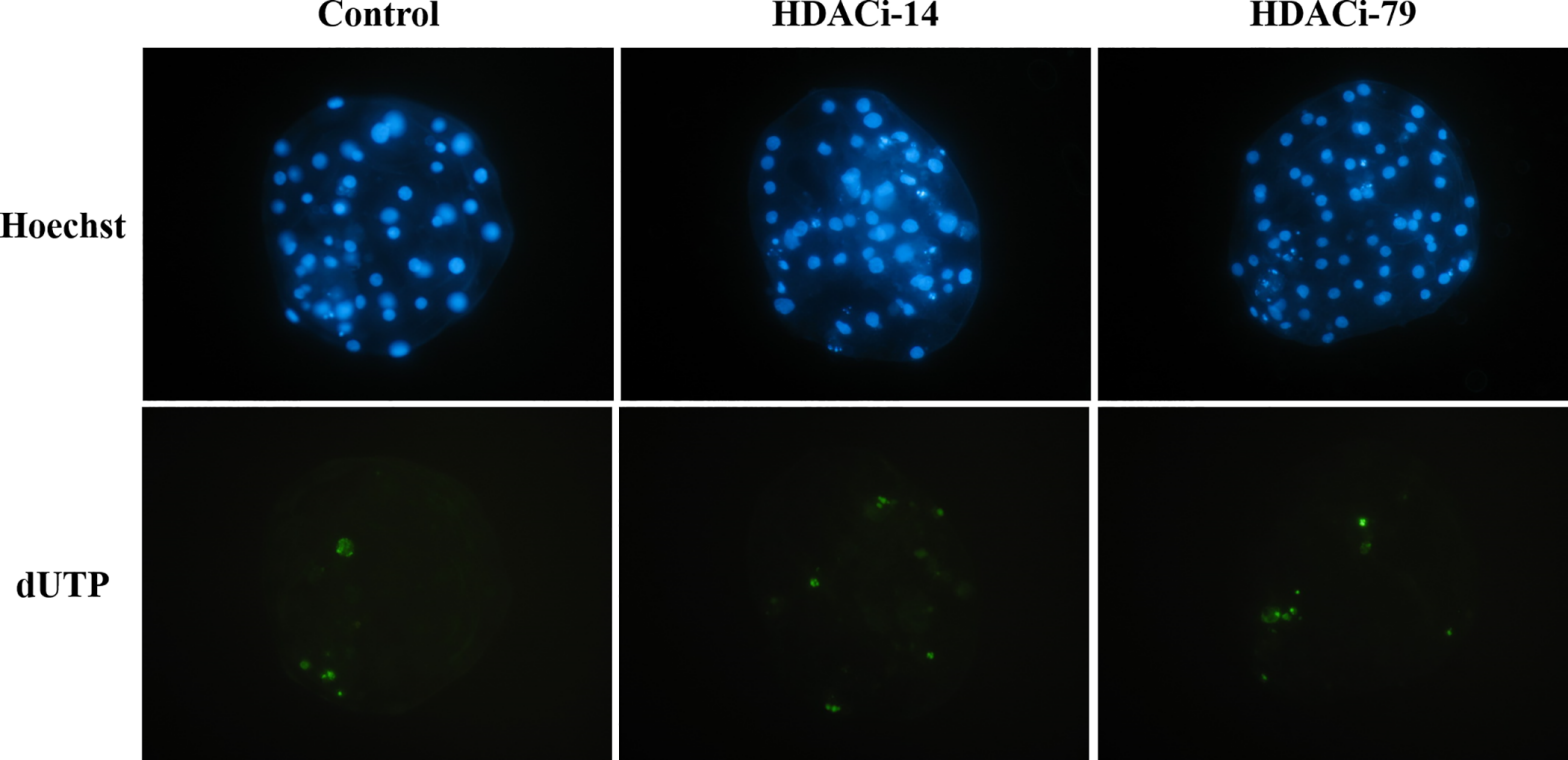
Apoptotic cells of blastocysts from different HDAC trreatments were detected by TUNEL assay. Hoechst staining for the total cell counts; dUTP: Green color represents apoptotic cells. No significant difference was observed among groups (P > 0.05).

The cloned embryos were evaluated for their developmental competence after being respectively treated with each HDACi, namely HDACi-14 or HDACi-79. There were no differences in cleavage rates between HDACi-14-treated and untreated embryos. When cloned embryos were treated with 2 μM HDACi-14, blastocyst rates were increased significantly (59.7%, *P <* 0.05; Table 2) compared to the Control (36.9%) and the 4-μM HDACi-14-treated groups (37.1%). However, the total cell numbers did not differ among the embryos treated with HDACi-14 and untreated ones. Additionally, supplementation of HDACi-14 at 1, 2, or 4 μM in the culture medium did not change the apoptotic indices of the cloned embryos (Table 2 and Fig 1).

**Table 2:** Developmental competence of cloned porcine embryos cultured with various concentrations of HDACi-14 (JWC-022214).

HDACi-14, μM	Number of embryos	Cleavage rate, % (n)	Blastocyst rate, % (n)	Total cell number	Apoptotic cells, %
0	65	93.9 (61)	36.9 (24)^b^	57	8.7
1	64	96.9 (62)	51.6 (33)^ab^	63	8.8
2	62	96.8 (60)	59.7 (37)^a^	66	8.0
4	62	91.9 (57)	37.1 (23)^b^	63	9.8

^a, b^ Numbers without the same superscripts in the same column differ (*P <* 0.05).

Replicates = 3

The HDACi-79 treatments at the concentrations of 2 and 4 μM had no effect on the cleavage rates of cloned embryos when compared to that of the Control group; however, they had significantly increased (*P <* 0.05) blastocyst rates (79.4 and 74.2%, respectively) compared to the control group (50.0%). The total cell numbers and the apoptotic indices in cloned embryos showed no difference between the HDACi-79-treated and untreated groups (Table 3 and Fig 1)

**Table 3:** Developmental competence of cloned porcine embryos cultured with various concentrations of HDACi-79 (JWC-017379).

HDACi-79, μM	Number of embryos	Cleavage rate, % (n)	Blastocyst rate, % (n)	Total cell number	Apoptotic cells, %
0	64	95.3 (61)	50.0 (32)^b^	60	8.9
1	64	95.3 (61)	68.8 (44)^ab^	56	10.7
2	63	98.4 (62)	79.4 (50)^a^	60	10.6
4	62	98.4 (61)	74.2 (46)^a^	55	9.4

^a, b^ Numbers without the same superscripts in the same column differ (*P <* 0.05).

Replicates = 3

For both HDACi-14 and HDACi-79, 2 μM was the optimized concentration that significantly increased blastocyst rates in the above results. Therefore, this concentration (2 μM) of HDACi-14 and -79 was chosen for further experiments.

### Histone acetylation (acH3:Lys9/Lys14) profiles of the cloned embryos after HDACi-14 and HDACi-79 treatments

**Fig 2:**
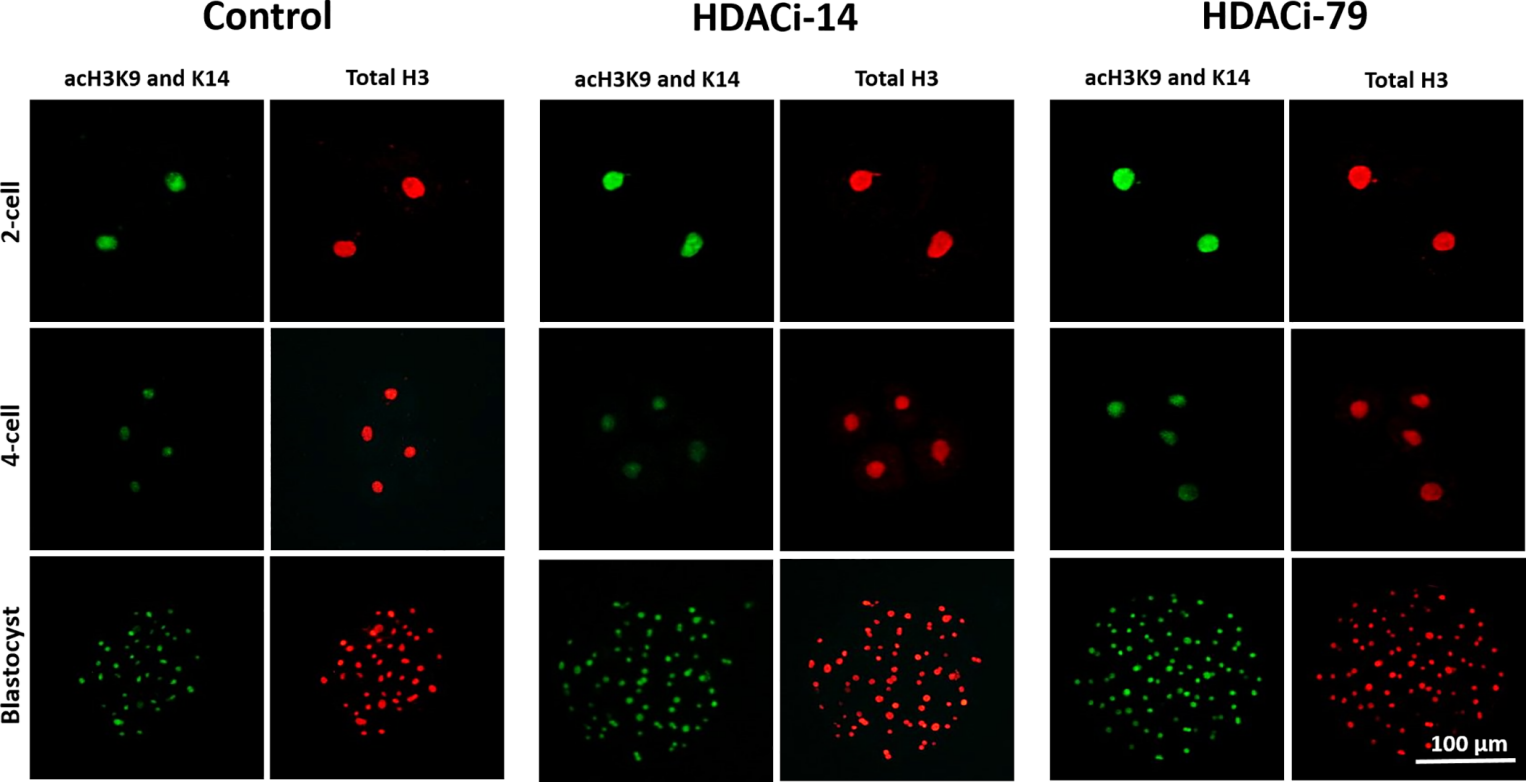
Immunocytochemical staining of histone acetylation (at acH3K9/K14) and total histone 3 (H3) in the cloned 2-celled (24 h after activation), 4-celled (48 h after activation), and blastocyst (168 h after activation) embryos cultured with HDACi-14 (2 μM) or HDACi-79 (2 μM). Scale bar = 100 μm.

**Fig 3:**
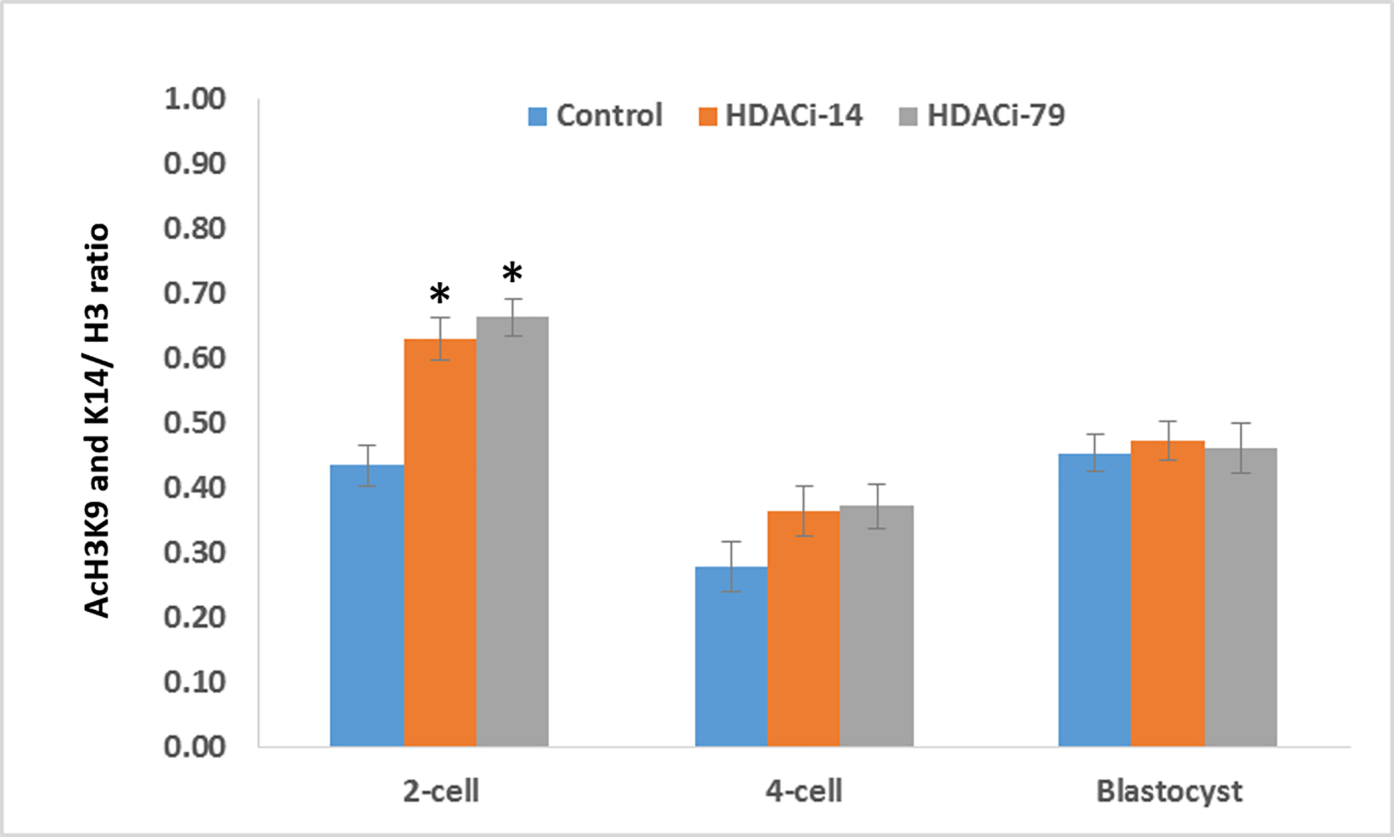
Ratios of nucleoplasmic fluorescence intensity of histone acetylation (acH3 K9/K14)/total histone 3 (H3) in cloned porcine embryos cultured in the medium supplemented with HDACi-14 (2 μM) or HDACi-79 (2 μM). * *P <* 0.05; four replicates.

Histone acetylation status of cloned embryos was examined by immunofluorescence staining (Fig 2 and Fig 3). At the two-cell stage, embryos treated with 2 μM of HDACi-14 or HDACi-79 had higher (*P <* 0.05) acetylation levels (ratios of acH3K9/K14:H3) than those of untreated embryos (0.63 and 0.66 *vs*. 0.43, respectively). However, ratios of acH3K9/K14:H3 of the 4-celled and blastocyst embryos showed no differences among treatment groups (*P* > 0.05).

### Effects of HDACi-14 and HDACi-79 treatments on gene expressions in cloned embryos

**Fig 4:**
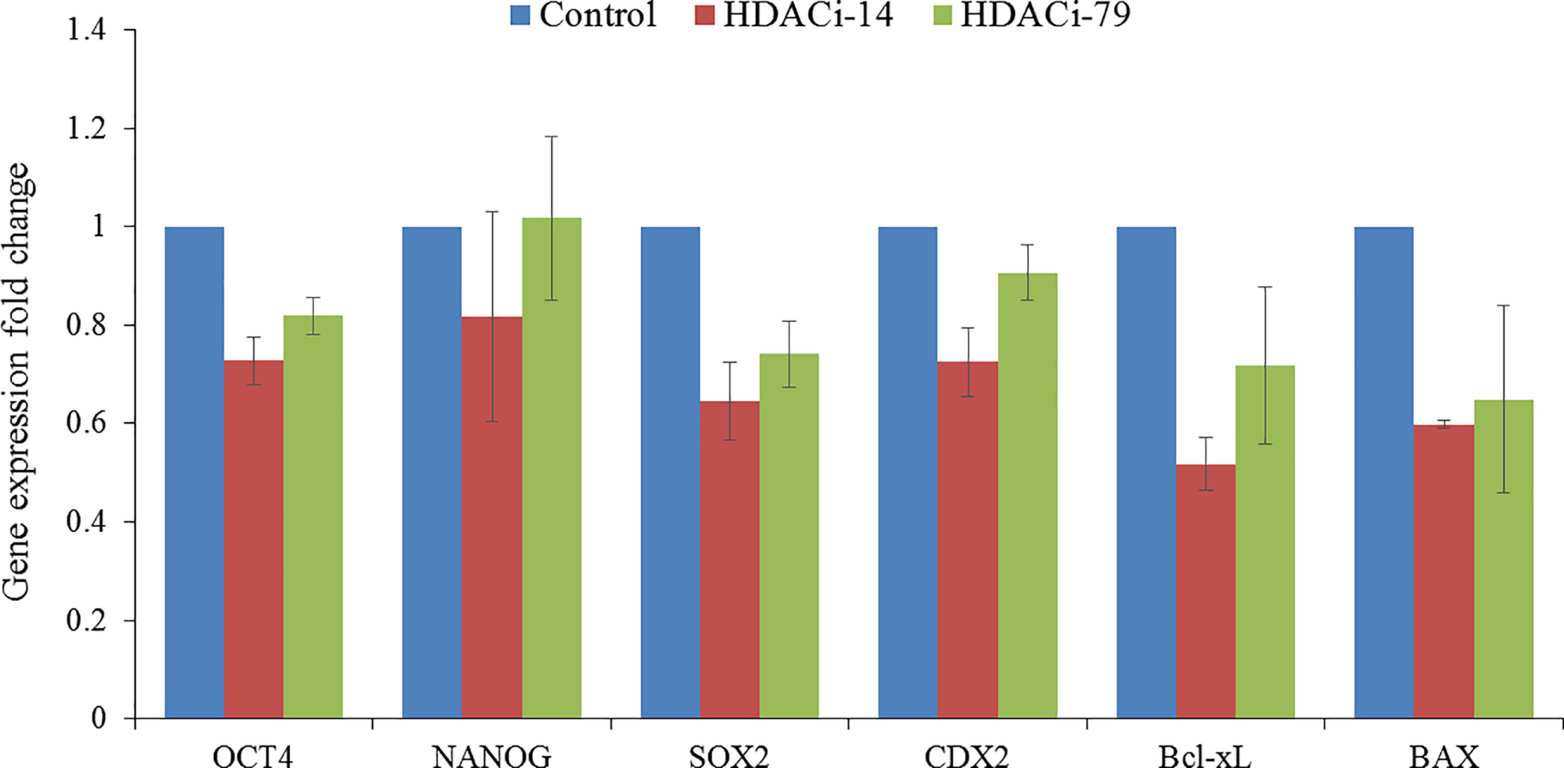
Expression of pluripotency-related genes (Oct4, Nanog, Sox2, Bcl-xL, Bax, and Cdx2) in cloned porcine blastocysts cultured in the medium supplemented with HDACi-14 (2 μM) or HDACi-79 (2 μM). Three replicates.

To test whether HDACi-14 or HDACi-79 supplementation in the culture medium affects the reprogramming efficiency, the expression levels of pluripotency-related genes (Oct4, Nanog, Sox2, and Cdx2) and apoptosis or survival-related genes (Bcl-xL and Bax) at the blastocyst stage (day 7) were studied using real-time PCR. Our results showed a trend that HDACi-79-treated blastocysts appeared to express pluripotency-related and survival-related genes slightly better than HDACi-14-treated blastocysts. However, no significant differences in the expression of abovementioned genes were detected among the Control and the treatment groups (Fig 4).

### Effects of HDACi-14 and HDACi-79 treatments on the derivation of porcine ntES cell lines from aggregated embryos

**Fig 5:**
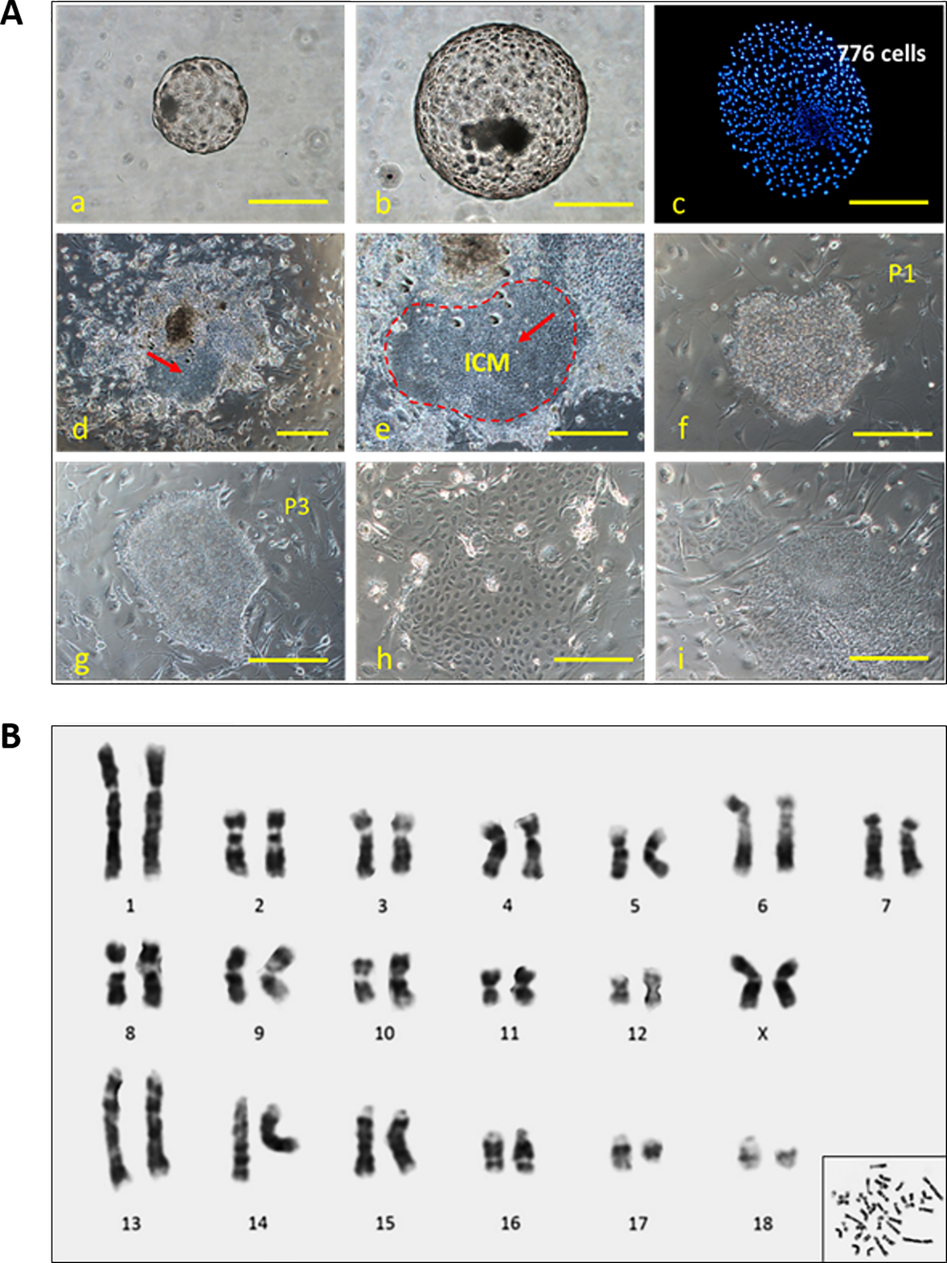
Panel A: Formation of ntES cell colonies derived from the blastocyst of the 3× cloned embryos. (a-c) Morphologies of non-aggregated, 3× aggregated blastocysts (day 7) and nuclear staining of 3× blastocysts by Hoechst 33342. (d and e) embryonal outgrowths with clear ICM cells (red arrow) after culture for 8–10 days. (f, g) Typical morphology of a porcine ntES cell colony (nt79-2) after passage. (h, i) Differentiated cells after passage. Panel B: G-banding karyotypes of porcine ntES-79-2 cells at passage 11. These cells have 18 pairs of autosomes and normal XX karyotypes. Scale bar = 100 μm.

The effects of HDACi-14 or HDACi-79 treatment on the establishment of ntES-like cells in 3× aggregated embryos are shown in Table 4 and Fig 5A. Four to six days after plating the aggregated embryos onto MEF layers, percentages of primary outgrowths from HDACi-79-treated cloned embryos formed were significantly higher (*P <* 0.05) than those from the HDACi-14-treated and the Control groups. Over the next 8 to 10 days, these colonies grew up to 2 to 5 mm in diameter without changing their morphologies. No differences were observed in the efficiency of primary colony formation among treatment groups. Although colonies from the HDACi-14 or HDACi-79-treated blastocysts appeared slightly larger compared to the Control group (*P* > 0.05). In addition, the numbers of ES cell lines that survived in further passaging were also not different among treatment groups. Percentages of primary colonies survived and continued to grow without apparent changes in their morphology beyond passage 3 in the HDACi-14, HDACi-79 and control groups, were 17.7, 25.0 and 14.3%, respectively. Cytogenetic analyses of the ntES cells (ntES-79-2 cells, passage 11) indicated that the cells examined were of normal karyotype without any sign of differentiation (2n = 38, Fig 5B).

**Table 4:** The effect of HDACi treatments to the 3× aggregated porcine embryos on the derivation of ntES-like cell lines.

Groups	Total No. of blastocysts	No. of primary outgrowths (%)	No. of primary colonies (%)	No. survived beyond passage 3 (%)
Control	14	6 (42.9)^b^	3 (21.4)	2 (14.3)
HDACi-14	17	7 (41.2)^b^	6 (35.3)	3 (17.7)
HDACi-79	20	12 (60.0)^a^	8 (40.0)	5 (25.0)

^a, b^ Numbers without the same superscripts in the same column differ (*P <* 0.05).

HDACi-14: 2 μM; HDACi-79: 2 μM; Replicates = 3

## Discussion

Previous studies have shown that TSA treatment improved *in vitro* and *in vivo* development of cloned embryos in mice, rabbits, bovine and pigs [3,17,19–22,35–37]. Recently, scriptaid had been verified by some studies; their results demonstrating that scriptaid treatment significantly enhanced blastocyst rates and increased total cells number per blastocyst in pig and buffalo cloned embryos [23,24,38]. In addition, Su et al. suggested that oxamflatin treatments efficiently improved the development of bovine SCNT embryos *in vitro* [39]. We therefore hypothesized that the newly synthesized HDACi (HDACi-14 and -79) also possessed the capacity to improve the development of cloned porcine embryos, as scriptaid, TSA and oxamflatin. To confirm this, the present study investigated the effect of two novel HDAC inhibitors on the development of porcine cloned embryos, the global histone acetylation patterns for nuclear reprogramming, and the derivation efficiency of ntES cell lines. We demonstrated that developmental competence of cloned embryos was improved by treatment with HDACi-14 or -79 when compared to that of the control embryo.

Although the underlying mechanisms of how HDACi-14 and -79 treatments improved the development of cloned embryos remains unclear, various clues had pointed to HDAC inhibition inducing hyperacetylation of the core histones, which resulted in the loosening of the chromatin into a transcriptionally permissive state to facilitate cell growth and proliferation [15,36]. The generally accepted mechanism proposed includes the re-expression of silenced genes and/or the silencing of downstream genes due to the regained access to the promoters by other modulatory factors [16]. As above mentioned, our novel HDAC inhibitors possess hydroxamic acid-like structure nomenclaturally categorized into the same group as TSA, scriptaid, SAHA, and oxamflatin, the inhibitors for Class I and IIa/b HDACs [40–44]; however these two novel drugs were specifically selective to the Class II HDACs, with only some minor activity to other Class of HDACs [33]. Conceivably, the beneficial effects and improved cloning efficiencies with these HDACi were most likely acting the same way as demonstrated in the previous study [25].

Some previous studies have indicated that TSA, one of the most commonly used HDACi, treatment significantly improved total and inner cell mass (ICM) cell numbers and ratios of ICM to trophectoderm (TE) cells in bovine blastocysts [45]. However, the total cell number in cloned porcine blastocysts was not improved after treatment with TSA [21,22]. Similarly, in the present study, no influence of HDACi-14 and -79 on the total cell number of cloned blastocysts was observed. Apoptosis is another major cause to the low cell viability and quality of the developing embryos [24,46,47]. Based on these results, TUNEL assays showed no difference in apoptotic cell indices in the HDACi-14 and -79-treated embryos compared to the untreated groups. It has been known that oxamflatin treatment reduced the apoptotic rate in bovine SCNT embryos, mainly because that oxamflatin suppresses expression of the pro-apoptotic gene Bax and stimulates expression of the anti-apoptotic gene Bcl-xL, which, in turn, enhances the development of SCNT embryos *in vitro* [39]. In contrast, TSA did not show beneficial effects on cell survival or apoptosis in bovine embryos, neither on the apoptosis of cloned porcine blastocysts derived *in vitro* [22,46]. Nevertheless, TSA, SAHA, and oxamflatin, but not VPA, have been shown to improve quality of cloned mouse embryos by reducing cell death of the ICM cells [25]. Therefore, even with the same HDACi, it appears that species-specific effects of these drugs do exist.

It has been well-known that histone acetylation-deacetylation is among the most studied epigenetic key events associated with gene silencing in the mammalian genome. In the present study, treatment of cloned embryos with HDACi-14 or -79 after nuclear transfer markedly increased levels of histone acetylation (acH3K9/K16) at the 2-cell stage. Consistently, our previous study also showed that TSA-treated cloned embryos had higher acetylation profiles at the 2- and 4-cell stage than those of the untreated embryos [22]. Moreover, increased level of histone H3 lysine 14 acetylation has been reported in TSA-treated cloned mouse embryos compared to the untreated embryos [26]. In addition, treatment of oxamflatin decreased the relative histone HDAC activity in cloned embryos and resulted in hyper-acetylation levels of histone H3 at lysine 9 (AcH3K9) and histone H4 at lysine 5 (AcH4K5) of the pronuclear stage, two-celled, and four-celled embryos partly through down regulating HDAC1 [48]. After being transferred into the recipient ooplasm, the donor nucleus of reconstructed porcine embryos had a lower histone acetylation level than that of their IVF counterparts at the pronuclear stage [17,36,49,50]. Increased histone acetylation levels on amino acid residues lead to a loose binding of nucleosomes to linker histones, i.e., relaxation of the chromatin structure to create a transcriptionally permissive condition [36]. Therefore, increased histone acetylation is associated with the accessibility of DNA replication protein complexes or 'DNA synthesomes' to the entire genome and facilitates cell proliferation [15]. In this study, we demonstrated that the abnormal histone acetylation pattern was corrected by HDACi-14 and -79 treatments. In this regard, HDACi-treated cloned embryos can reach a histone acetylation level comparable to that of IVF embryos [17].

Finally, we reported the use of HDACi treatments and embryo aggregation technique for the isolation of putative porcine ES cells from cloned embryos. We found that HDACi-14 and -79 increased the percentage of cloned embryos developing to the blastocyst stage, but their total cell numbers did not increase. However, we have previously shown that the total cell numbers of the whole embryo, ICM, and ICM/TE ratios were increased by embryo aggregation [30]. As demonstrated in this study, more proportions of primary outgrowths were obtained from HDACi-79-treated 3×aggregated embryos (60.0%) compared to the HDACi-14-treated and the untreated groups (41.2% and 42.9%, respectively). Moreover, a slightly better efficiency (25% by numbers of ES cell colonies grown beyond passage 3) in ES cell line establishment was derived by the HDACi-79 treated group. Overall, there is a lack of reports on pig ntES cells generated by HDACi-treated embryos. Recently, TSA was the most extensively used inhibitor for improving the efficiency of ntES cell derivation in pigs, mice and humans [8,9,20,51]. This may be due to the drugs reversibly and specifically inhibiting histone deacetylases, and results in hyperacetylation of core histones that modulate chromatin structure. The increase in histone acetylation promotes selective gene transcription and associated with the improved cell number and quality of cloned blastocysts [17–25], which, in turn, lead to the increased efficiency of ES cell establishment. In this study, we have added two more newly synthesized HDAC inhibitors to the list.

In conclusion, we clearly demonstrated that, by treating cloned porcine embryos with optimized concentrations of novel HDACi, they evidently acquired their competency of *in vitro* development during early embryogenesis. In other words, these new drugs can improve the developmental competence of cloned embryos up to the blastocyst stage. Due to acting positively through the epigenetic level, our novel HDACi may also maintain or enhance histone acetylation during early embryogenesis of cloned pig embryos, from which putative porcine ntES cell lines can be efficiently isolated.

## Supporting information

S1 Checklist ARRIVE guidelines checklist. (PDF)

S1 Data All raw data used for quantification in this work. (XLSX)
